# An Automated Method to Quantify Microglia Morphology and Application to Monitor Activation State Longitudinally In Vivo

**DOI:** 10.1371/journal.pone.0031814

**Published:** 2012-02-28

**Authors:** Cleopatra Kozlowski, Robby M. Weimer

**Affiliations:** Department of Biomedical Imaging and Department of Neuroscience, Genentech, Inc., South San Francisco, California, United States of America; Emory University, United States of America

## Abstract

Microglia are specialized immune cells of the brain. Upon insult, microglia initiate a cascade of cellular responses including a characteristic change in cell morphology. To study the dynamics of microglia immune response in situ, we developed an automated image analysis method that enables the quantitative assessment of microglia activation state within tissue based solely on cell morphology. Per cell morphometric analysis of fluorescently labeled microglia is achieved through local iterative threshold segmentation, which reduces errors caused by signal-to-noise variation across large volumes. We demonstrate, utilizing systemic application of lipopolysaccharide as a model of immune challenge, that several morphological parameters, including cell perimeter length, cell roundness and soma size, quantitatively distinguish resting versus activated populations of microglia within tissue comparable to traditional immunohistochemistry methods. Furthermore, we provide proof-of-concept data that monitoring soma size enables the longitudinal assessment of microglia activation in the mouse neocortex imaged *via* 2-photon *in vivo* microscopy. The ability to quantify microglia activation automatically by shape alone allows unbiased and rapid analysis of both fixed and in vivo central nervous system tissue.

## Introduction

Microglia are the native innate immune cells of the central nervous system. In response to insult, they become activated, migrate to sites of damage, phagocytose cellular debris, and release cytokines [1]. This response is thought to rapidly mitigate local infection and cell damage. However, chronic activation of microglia has been implicated as a causative factor in a range of neurological disorders [2]. The dichotomy of microglia biology in health and disease is poorly understood due, in part, to a lack of methods to efficiently quantify microglia activation longitudinally in the context of their cellular environment.

Mechanical, metabolic or inflammatory activation of microglia results in several characteristic physiological changes within individual cells. Microglia undergo a shift in gene expression profile [3], [4], [5]; Notably, expression of the calcium binding protein IBA-1 [6] and the lysosomal protein CD68 [7] are up-regulated. Consequently, immunohistochemistry detection of IBA-1 and/or CD68 is routinely used to assess activation within tissue. However, histological approaches do have limitations, for example they are incompatible with the longitudinal assessment of individual microglia *in situ*.

Another characteristic activation-induced change in microglia is a transformation in cellular morphology [1]. At rest microglia exhibit a ramified cell morphology with numerous thin processes extending tens of microns away from their soma. Upon activation, these thin processes are drawn back into their soma, resulting in a rounded amoeboid-like appearance. Recent work suggests that this change in morphology may provide an opportunity to quantitatively study microglia activation within their native cellular context longitudinally.

Utilizing time-lapse imaging of FITC labeled microglia within neonatal rat brain slices, Stence and colleagues demonstrated that microglia undergo a stepwise change in cell morphology with activation [8]. Specifically, mechanical activation induced the retraction of fine processes on timescales of minutes prior to changes in cell motility. Measures of processes length, therefore, enabled the quantification of activation response on a per cell basis.

The use of morphological change as a readout of microglia activation is further supported by studies comparing cell morphology to the expression level of known markers of microglia activation. P2Y12 is a metabotropic purinergic receptor down-regulated within microglia under activating conditions [9]. By comparing the expression level of P2Y12 after mechanical insult to the number of primary processes emanating from a microglia, Haynes and colleagues demonstrated a positive correlation (R^2^ = 0.93) between process number and P2Y12 expression, consistent with microglia converting to an amoeboid shape upon activation.

While informative, the studies described above relied on manual measurement of microglia morphology. Manual methods are time consuming, susceptible to human bias, and therefore difficult to scale up to analyze larger datasets. To circumvent these limitations, we sought to develop automated image analysis methods to segment the cell shape of individual microglia from fluorescence micrograph datasets, calculate morphological parameters that characterize individual cell shape, then quantitatively assess which parameter best reports changes in microglia activation state.

Here, we report the implementation of an iterative thresholding method to segment the cell shape of individual microglia with ∼90% accuracy compared to manual methods, yet that requires ∼1/100th the time, and no human intervention. Segmentation relies on *a priori* knowledge of a single characteristic of microglia: cell size. Utilizing this automated segmentation method in parallel with immunohistochemistry staining for known markers of microglia activation, we demonstrate that automated measures of microglia morphology can be used to quantify their activation state in tissues, on a per cell basis. We find that soma size best correlates with expression level of IBA-1 under activating conditions. Finally, in a proof-of-concept study we illustrate the utility of tracking soma size longitudinally as a quantitative metric of microglia activation *in vivo.* The ability to automatically track microglia activation without antibody staining will expedite the study of microglia biology and their role in disease.

## Materials and Methods

### Ethics Statement

All procedures were carried out with Institutional Animal Care and Use Committee (IACUC) approval in accordance with the institution's ethical guidelines (Genentech Protocol Numbers: 2-photon intravital imaging 08-1737, immunohistochemistry 10-1389).

### Animals and treatment conditions

Two to four-month old CX3CR1-EGFP heterozygous C57BL/6 animals [10] were used for all experiments. Microglia were activated *in vivo* by I.V. injection of 1, 2 or 4 mg/kg of Escherichia coli LPS (serotype EH100 Sigma) dissolved in 0.9% saline. These mice were euthanized after 24 h or 48 h (as indicated) for histological analysis. An independent set of animals were examined *in vivo* by 2-photon microscopy through a cranial window, ± LPS treatment or laser ablation, and imaged up to 96 h.

### Confocal Microscopy of ex vivo tissues

Coronal sections of 40-µm thickness were prepared on a cryostat and mounted onto slides (see **I**mmunohistochemistry). Cortical regions of the coronal sections were examined using an inverted laser scanning confocal microscope (SP5 Leica with multi-immersion objective 20×0.7 NA), with excitation lasers at 488 nm and 633 nm to excite EGFP (bandpass 510/20 nm) and Alexa 647 (bandpass 675/25 nm), respectively. The laser was then tuned to 533 nm to excite Alexa-564 (bandpass 615/30 nm), to avoid bleed-through of EGFP into the Alexa-564 channel. Two regions per mouse, covering 776×776 µm, ∼10 optical sections 1 µm apart (0.76 µm/pixel resolution in X and Y), were imaged with three line averaging to reduce noise.

### Generating maximum intensity projections

All segmentation and quantification was performed on 2D mean intensity projections (MIPs) of 3D image data. For fixed samples, the top 10 µm were used to create MIPs from cortical regions. The imaged depth approximately corresponds to the antibody penetration depth. For *in vivo* data, image volumes of 100 µm axial depths were obtained. Sub-volumes of 10 µm thickness were used to create MIPs, separated by 20 µm gaps, resulting in three MIPs analyzed per imaged volume. As soma size is approximately 10 µm in diameter, we reasoned that the 20 µm gap between volumes used for maximum projections eliminated the possibility of the same cell being identified within multiple MIPs.

### Image segmentation

All morphometric segmentation was based on green fluorescence intensities generated from imaging EGFP expressing microglia of CX3CR1-EGFP transgenic mice. The automated segmentation routine consists of three steps:

First, the positions of individual cells within a field of view were determined by: 1) identifying objects consisting of connected pixels with intensity values above a regional maxima intensity value (imregionalmax function within MATLAB), then 2) calculating the centroid for each object detected with a summed pixel area greater than 50 µm^2^. Each centroid defined an individual microglia cell positions (CP).

Second, cell masks (CM) were generated per CP *via* iterative local threshold segmentation of EGFP+ pixels. Specifically, within a 120 µm×120 µm local region (LR) centered on each CP, candidate cell masks (CCM) were generated by identifying connected pixels with intensity values greater than the threshold calculated by Otsu's method [11] within the LR. Otsu's method identifies a threshold pixel value that minimizes the interclass pixel intensity variance between the two pixel classes (‘on’ or ‘off’) within the image (graythresh function within MATLAB). The summed pixel area within a CCM is compared to the predetermined variable, target cell size (TCS, discussed further in *Target cell size*). If the CCM equals the TCS ± 100 µm^2^ the mask is considered to be an accurate CM for the associated CP. If not, the threshold is adjusted by a quantity proportional to the difference between the current CCM size and TCS averaged over every past iteration (Figure S1). The iteration stops when either the CCM is within an accepted range of the TCS ± 100 µm^2^ or if the size of the CCM stabilizes to a fixed value for more than two consecutive iterations at which point the mask is considered to be an accurate CM for the associated CP. This averaging method ensures that the calculations converge.

Third, each CM is subject to two subsequent tests to confirm it accurately represents a single microglial cell. Test 1: Does the CM touch the boundary of the LR, thus potentially misrepresent the area of the cell? If so, the CP and CM are removed from further analysis. Test 2: Does the CM contain a single soma? A cell soma mask (CSM) is defined as a contiguous area larger than 16.7 µm^2^ within the CM with pixel values 50% greater than the rest of the CM. If the CM contains more than one CSM it is excluded from further analysis as it likely represents a dividing cell or two cells in close proximity that could not be accurately resolved. If a CM does not contain a CSM it is also discarded, as it likely represents a microglia positioned above or below the imaged volume.

### Morphometric measures of cell shape

At rest microglia exhibit a ramified morphology, yet become amoeboid upon activation. To quantitatively describe this morphological switch we defined a series of morphological parameters expected to capture this change. These include cell perimeter length, the length around the periphery of each cell; cell spread, the average distance from the cell center of mass to its detected extremes; eccentricity, the ratio of the major axis to the minor axis of the smallest circle that can fit the extensions of the cell; roundness, 4π×area/cell perimeter length^2^; soma size, the area contained within the soma mask. All parameters were calculated from individual segmented soma or cell masks (see *Image segmentation*) using the MATLAB regionprops function with the properties specified as: perimeter, extrema, eccentricity and area, respectively.

### Immunohistochemistry and automated quantification of protein expression per cell

Animals were euthanized by CO_2_ asphyxiation and immediately transcardially perfused with PBS followed by 10% sucrose 4% paraformaldehyde in PBS. Whole brains were placed in the same fixative overnight and then transferred into 30% sucrose. Coronal sections of 40 µm thickness were prepared on a cryostat and mounted onto slides. Sections were permeabilized (0.1% Triton in PBS), blocked 1 h at room temperature (5% BSA 0.3% Triton in PBS), immuno-stained with 1∶1000 rabbit anti-IBA-1 (Wako Chemicals) or 1∶500 rat anti-CD68 (Serotec) overnight at 4°C. Sections were washed (0.1% Triton in PBS) and secondary antibodies 1∶1000 anti-rabbit Alexa-564 and anti-rat Alexa-647 (Invitrogen Molecular Probes), respectively, were applied for 2 h at room temperature. Sections were mounted with VectaShield (Vector Labs).

Similar to cell shape segmentation, quantification of immunofluorescence data can be complicated by inhomogeneity of fluorescence intensities across a large field of view. Often, fluorescence intensities are quantified by measuring overall image brightness, and subtracting the mean background fluorescence. Since our work is concerned with fluorescence intensities in individual microglia, each cell was analyzed within its local region of interest as defined during image segmentation (Figure S5). IBA-1 or CD68 expression was quantified within each cell mask based on secondary antibody fluorescence intensity. The mean fluorescence intensity was divided by the mean background intensity within LR. The mean background intensity was calculated from the mean intensity within the LR excluding the cell mask. This quotient method was used instead of a subtractive method, as the former provided more consistent results with tissues of varying staining intensities.

### Target cell size (TCS)

Our segmentation routine utilizes a single variable, TCS, to minimize the impact of local fluorescence intensity variability in segmenting individual cells, and thus can potentially influence the values calculated for the parameters characterizing microglia morphology. Initially, this value was estimated based on the approximate size of individual microglia when represented in MIPs of 10 µm volumes 700×700 µm^2^ XY fields of view. Once the segmentation routine, morphological parameters and immunostaining techniques were established, we further explored the influence of the TCS on the ability to distinguish LPS-induced microglia activation from control (Figure S2). We found that a TCS of 500±100 µm^2^ gave the highest number of detected CMs and highest differentiation between activated and resting microglia by both morphological and marker expression matrices. Furthermore, the value of 500 µm^2^ is consistent with previous estimates of microglia size in the CNS [12]. Note that if the target cell size is set too high, it will detect not only cells, but also neighboring touching cells. If it is set too low, its ability to distinguish activated and resting cells diminish, as the main delineating factor is expected to be cell process extensions. For all analysis in this text, TCS was therefore set to 500 µm^2^.

### 2-photon *in vivo* microscopy

Cranial windows were implanted above the somatosensory cortex as described previously [13]. Briefly, animals were anesthetized using isoflurane, secured on a stereotax, and a craniotomy was performed to remove approximately a 2-mm diameter region of skull leaving the dura intact. A custom-made 3-mm diameter No. 1 glass cover slip was placed above the exposed tissue and secured to the remaining bone with dental acrylic. A metal bar was embedded within the dental acrylic next to the glass and used to secure the mouse at a fixed angle during imaging. Mice were allowed to recover a minimum of two weeks before beginning an imaging experiment.

For each imaging time point, mice were anesthetized with 1–2% isoflurane and injected I.V. with 100 µl of the vascular marker AngioSense 680 (VisEn Medical). Animals were head restrained at the stage of a 2-photon microscope equipped with four independent photomultiplier tubes (Prairie Technologies UltimaIV) and powered by a tunable MaiTai DeepSee Ti-sapphire laser (Spectra-Physics). Images were acquired using a 40× N.A. 0.8 objective (Olympus), 910 nm excitation wavelength, 440/80 nm, 510/60 nm and 705/50 nm bandpass filters for second harmonic detection of the dura, EGFP+ and AngioSense 680, respectively. Laser power and dwell time were constant within an experiment (∼20 mW back focal plane power, 4.8 µs/pixel, respectively). Only those areas between 50 µm and 200 µm below the dura were used for imaging. For the first imaging session, up to 3 neighboring 300×300×100 µm (XYZ) areas were imaged (512×512 pixels spaced 1 µm in Z, 0.58 µm/pixel resolution in X and Y). For subsequent imaging sessions, imaging areas were identified *via* the unique patterning of the vasculature. Note: daily I.V. administration of AngioSense, a macromolecular fluorescent probe with relatively slow systemic clearance, resulted in a gradual increase in red fluorescence within the vasculature throughout the longitudinal studies.

### Statistics

Statistical analysis was performed using the MATLAB Statistical Package. Results are shown as mean values ± standard deviations (STD). Statistical significance is calculated by Student's T-test, and indicated as follows: *p<0.05, **p<0.01, and ***p<0.001. Correlation was calculated by Pearson's Correlation Formula.

## Results

### Automated segmentation of fluorescently labeled microglia

At rest microglia exhibit a ramified morphology: thin processes extending tens of microns radial from a larger cell soma. This shape provides unique challenges for automating cell segmentation from fluorescent micrographs using standard global threshold methods. For example, subtle inhomogeneities in fluorescence excitation or detection across a field of view can influence fluorescence intensity values across an image and even a single cell. In the context of fluorescently labeled microglia this translates to errors when segmenting thin processes. To circumvent this problem we developed an iterative local threshold method, which enables the segmentation of microglia within tissues at accuracies comparable to manual segmentation (see Materials and methods).

**Figure 1:**
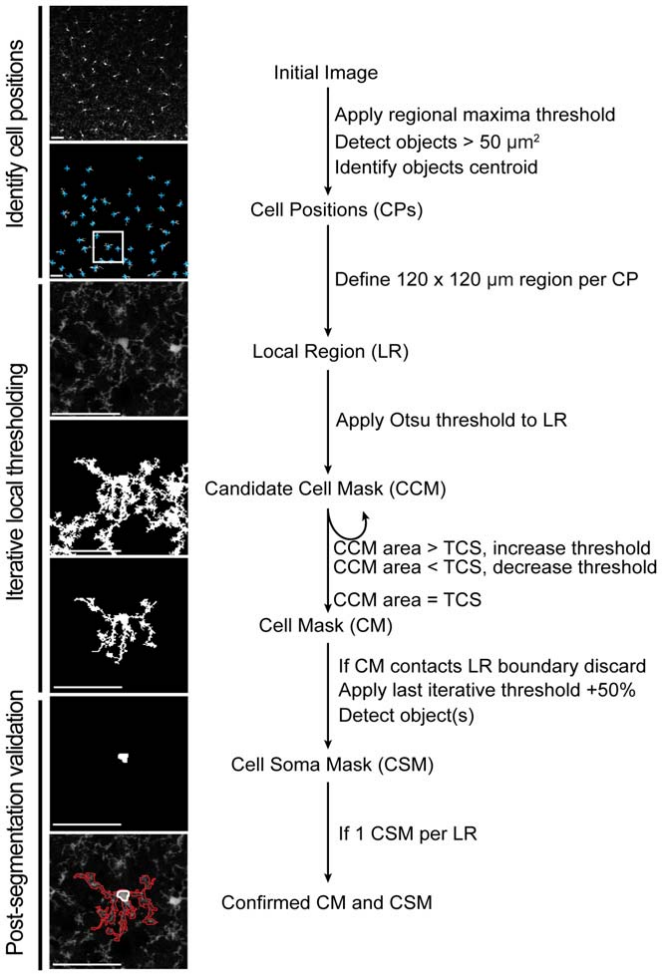
The image processing strategy for segmenting cell shape of individual microglia. Cell positions (CP, blue cross) within a maximum intensity projection were identified by a local maxima-finding algorithm. Within a 120×120 µm local region (LR, white square) centered per CP, cell masks (CM) are segmented through an iterative local threshold algorithm. For each CM, a cell soma mask (CSM) is defined as an object with pixel values greater than the last iterative threshold+50% and contiguous area greater than 16.7 µm^2^. A CM (red outline) and CSM (white outline) are considered accurate representations of a cell (bottom figure) if the CM is not touching the edge of the LR boundary and has a single CSM. Scale bar equals 50 µm.

Briefly, our segmentation strategy relies on three steps (Figure 1). 1) The identification of microglia cell positions (CP) within a field of view, achieved through a regional maxima finding algorithm. 2) Applying an iterative threshold segmentation routine within the local region surrounding each detected CP to generate an accurate cell mask (CM) (Figure S1 and Figure S2). 3) Post-segmentation testing to verify that each CM accurately represents a single cell with a single soma. Furthermore, since segmentation does not directly relate to fluorescence intensities, the above segmentation approach is relatively insensitive to pixel saturation.

When implemented in MATLAB, the computing time per cell within a field of view is approximately 0.2 seconds. Overall, 70 to 100% of cells detected by manual segmentation methods are correctly identified by this automated algorithm (Figure S3). Note that due to their ramified morphology, processes from resting cells are more likely to touch neighboring cells, thus fewer resting cells are properly segmented per area compared to activated cells. Similarly, conditions that result in microglia aggregation may result in fewer detected cells as their probability of contact increases with increasing proximity.

### Per cell morphometric characterization of microglia within tissue

**Figure 2:**
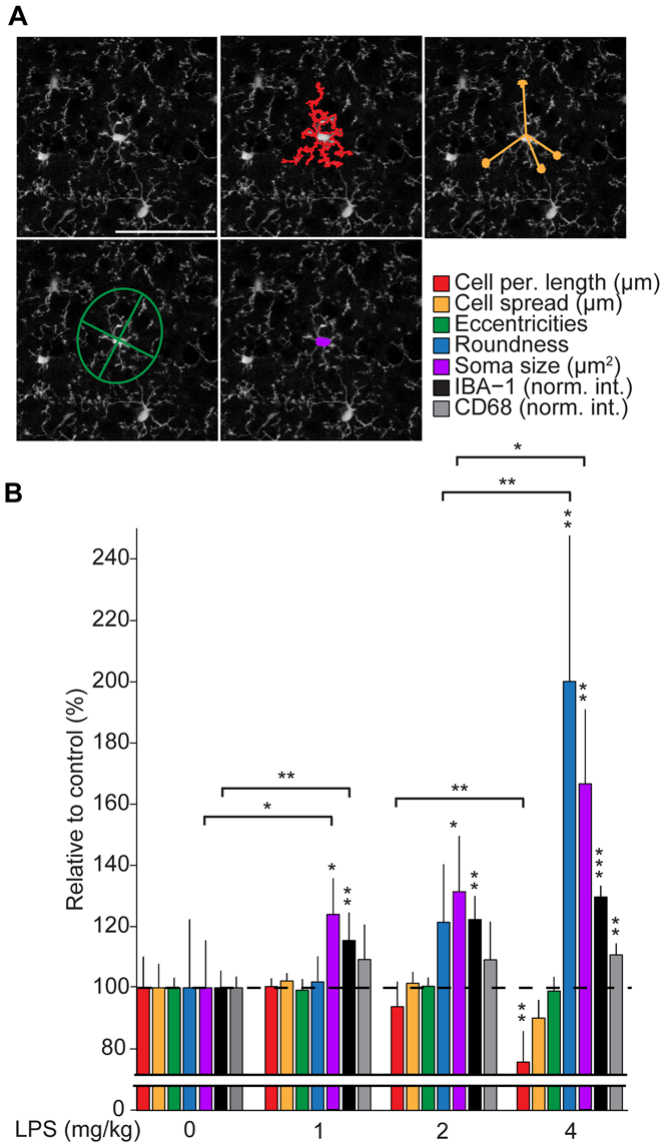
Morphometric parameters that quantitatively capture LPS-induced microglia morphology change. (**A**) Morphological parameters defined to characterize the shape of segmented cells: cell perimeter length, cell spread, eccentricity and soma size. Scale bar equals 50 µm. (**B**) Relative change in microglia morphometric parameters, IBA-1 and CD68 expression as a function of LPS dose. For each LPS condition, a mean value for each morphological parameter is calculated per animal, which is then normalized to the average value for that parameter in the control group (0 mg/kg LPS). This relative value per animal is then used to calculate a mean value and standard deviation per condition, as plotted, n = 7, 6, 6 and 5 animals/condition, respectively. Asterisks indicate statistical significance compared to 0 mg/kg LPS (see Materials and Methods), those over horizontal lines indicate statistical significance between conditions.

Once segmented, each cell mask provides the spatial coordinates of individual microglia. To quantitatively describe their shape, we defined a series of morphometric parameters designed to capture the ramified morphology of resting microglia (Figure 2A), see Material and Methods. These include cell perimeter length; cell spread, which is analogous to the ‘process length’ metric from previous studies [8]; eccentricity; roundness, which is similar to the morphology quantification used by Haynes et al. [9]; and soma size. Once implemented into the image analysis routine post-segmentation, these morphological parameters could be automatically calculated per cell *en masse* ∼100 times faster than equivalent manual methods (Figure S3). Within populations of cortical microglia cell perimeter length, cell spread and soma size exhibit a normal distribution with mean values of 468±89 µm, 29.7±7.7 µm, 27.7±4.5 µm^2^, respectively (Figure S4). Roundness and eccentricities, conversely, exhibit skewed distributions with median values of 0.0283 (first quartile = 0.0218, third quartile = 0.0321) and 0.787 (first quartile = 0.716, third quartile = 0.892), respectively.

### Morphometric-based quantification of microglia activation

Upon activation, microglia undergo a characteristic morphological transformation in which they take on an amoeboid-like shape. To test if our automated segmentation and morphometric analysis methods enable quantitative assessment of this activation-induced morphological switch, we compared the calculated values as a function of LPS-induced activation condition. LPS induces microglia activation through the TLR4 pathway; Systemic application of LPS is commonly used to activate these cells *in vivo*.

CX3CR1-EGFP heterozygous animals were injected I.V. with varying doses of LPS (0, 1, 2, and 4 mg/kg) 24 h prior to collection. For each LPS condition, the calculated morphometric parameters were normalized to control condition (0 mg/kg) values and plotted as a percentage, where the mean values in the control sample are defined as 100% (Figure 2B). Strikingly, several parameters exhibited a significant change as a function of injected LPS dose: roundness and soma size increased (roundness: 106±9%, 126±19% and 213±44%; soma size: 124±12%, 133±17% and 170±29% for 1, 2 and 4 mg/kg doses of LPS, respectively; mean ± STD) while cell perimeter length decreased (98±3%, 92±8% and 74±10% for 1, 2 and 4 mg/kg doses of LPS, respectively; mean ± STD). Soma size was the only parameter tested that exhibited a significant change compared to control over the entire LPS dose range.

To confirm systemic LPS injection induced microglia activation over the dose range, we examined condition-dependent expression of IBA-1 and CD68, as expression of both proteins increase in activated microglia [6], [14]. Cortical sections from CX3CR1-EGFP heterozygous animals were immunostained for IBA-1 and CD68 using red and infrared fluorophore linked secondary antibodies, respectively, and then imaged to detect green, red and infrared fluorescence. Cell masks, identified through segmentation of EGFP signal, were used as cell-specific regions of interest for quantifying immunofluorescence within individual microglia (Figure 2B and Figure S5). With LPS treatment both IBA-1 and CD68 detection increased within cells (IBA-1: 115±9%, 122±8% and 130±3%; CD68: 109±11%, 109±12% and 110±4% for 1, 2 and 4 mg/kg LPS injected, respectively; mean ± STD), however only IBA-1 exhibited a significant increase over the entire LPS dose range. The concurrent dose-dependent increase in IBA-1 expression and measured change in cell shape, specifically soma size, suggests that morphometric analysis of microglia proves a quantitative method for assessing microglia activation *in situ.*

**Figure 3:**
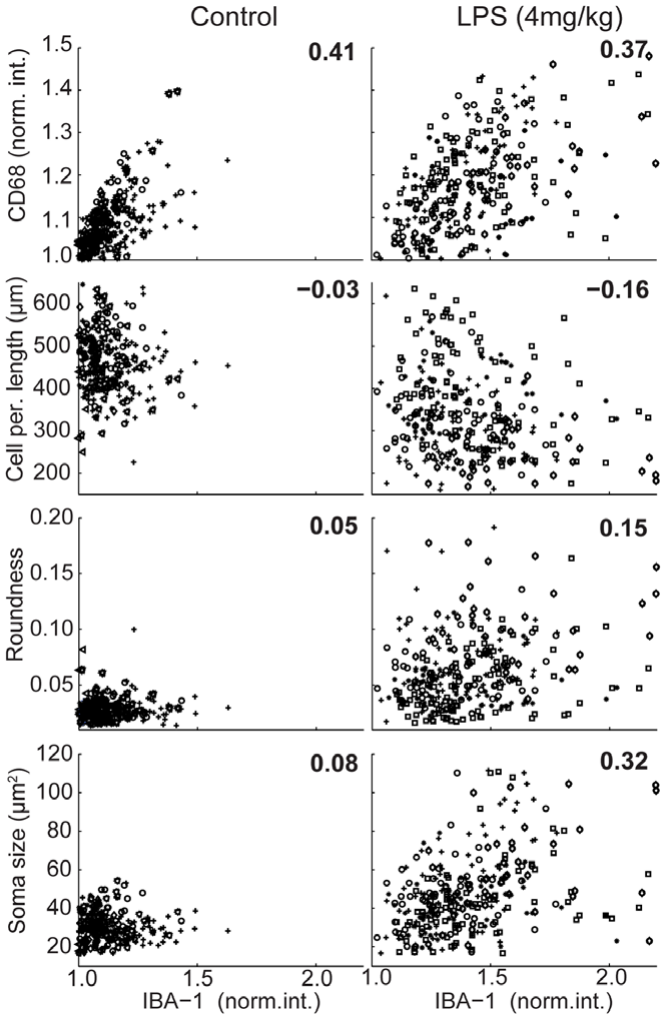
Correlation between morphological parameters and IBA-1 expression. Correlation plots between IBA-1 expression and CD68 expression or the various morphometric parameters, under control and 4 mg/kg LPS conditions (n = 7 and 5 animals/group, respectively). Cell populations from individual mice are plotted in different symbols. The numbers indicates the mean linear correlation coefficient (R^2^) value per comparison derived from linear fits to each animal dataset within a condition.

The ability to quantify cell shape and protein expression level on a per cell basis within a large population provides an opportunity to explore the relationship between cell morphology, gene regulation and LPS-induced activation. We tested if our automated measurements are capable of revealing per cell relationships by comparing the detected expression level of IBA-1 versus CD68 under the various LPS condition (Figure 3 and Figure S6). In all conditions, IBA-1 and CD68 expression were correlated (mean R^2^>0.3); however, there is significant heterogeneity within any given population. Even at the highest LPS dose, subsets of cells exhibited low expression of IBA-1 and CD68, suggesting that not all microglia activate *in vivo* with systemic injection of LPS.

We extended this analysis to test which, if any, morphological parameters correlate with IBA-1 expression on a per cell basis. Within a given LPS condition, cell perimeter length, roundness, and soma size correlated with each other (data not shown). However, only soma size exhibited a per cell correlation (mean R^2^>0.3) with IBA-1 expression in multiple LPS conditions (Figure 3 and Figure S6), further supporting the use of soma size as a morphological surrogate to assess microglia activation *in situ*.

### Longitudinal measure of microglia activation in vivo

**Figure 4:**
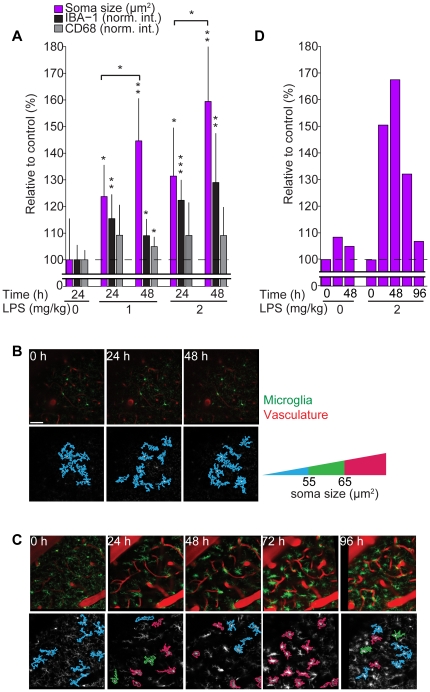
Longitudinal assessment of microglia activation *in vivo* by tracking changes in soma size. (**A**) Quantification of soma size, IBA-1 and CD68 expression relative to control conditions 24 or 48 hours post LPS injection. Asterisks indicate significance for each condition compared to control (0 mg/kg LPS, 24 h), horizontal line represents statistical comparisons between indicated time points, n = 5, 6, 6, 6 and 6 animals/group, respectively. (**B** and **C**) Top: representative images of microglia (green) and blood vessels (red) imaged *in vivo* at indicated time points. Bottom: Outlines of segmented microglia from the corresponding image above color-coded to indicate soma size per cell under control (**B**) and LPS (2 mg/kg) (**C**) conditions; red corresponds to microglia with soma size >65 µm^2^, thus activated. (**D**) Quantification of soma size measured *in vivo* as a function of time and LPS dose. Data from one control animal, and one LPS stimulated animal, are shown. Scale bar equals 50 µm.

The correlation between soma size and expression of microglia activation markers suggests this morphological feature may provide a method to monitor microglia activation longitudinally within native tissue. To test this, we quantified soma size and the expression of IBA-1 and CD68 as a function of time from cohorts of CX3CR1-EGFP heterozygous animals dosed 1 mg/kg or 2 mg/kg LPS 0, 24 or 48 h prior to tissue collection (Figure 4A). Soma size, as well as IBA-1 and CD68 expression, exhibited time-dependent increases most pronounced in the 2 mg/kg dose, suggesting that the time course of LPS-dependent microglia activation could be assessed by monitoring soma size.

To test this possibility further, we conducted proof-of-concept *in vivo* imaging studies. First, we tested if differences in imaging properties across microscopes, such as pixel size and optical section thickness, significantly influences estimates of soma size. Varying either parameter did not influence calculated values (Figure S7). We then longitudinally imaged microglia *in vivo* through a cranial window by 2-photon microscopy. In control, microglia were imaged daily for 3 consecutive days (Figure 4B), no change in soma size was detected (Figure 4D). Injection of 2 mg/kg LPS, however, induced a time-dependent change in soma size: a 2-fold increase over the initial 48-hour time period, with similar timing and magnitude as measured *ex vivo*, before subsiding to near resting levels by 96 hours (Figure 4C and 4D). Similar time-dependent changes in microglia soma size were observed following I.P. injection of LPS across a population of animals enabling quantitative assessment of microglia activation/resolution (Kozlowski and Weimer, unpublished), thus supporting the use of the image analysis routine outlined here in quantifying microglia activation dynamics.

To test if soma size could be used to track microglia activation under conditions other than LPS stimulation, we utilized focal thermal damage to activate microglia. Small (5×5×5 µm) cortical regions were targeted for laser ablation and associated areas imaged over four days. Within three cortical regions, targeted focal ablation induced an increase in the average soma size of nearby microglia. In adjoining regions away from the ablation site, microglia soma size remained unchanged (Figure S8). Taken together, these data suggest that monitoring the change in soma size provides a longitudinal readout of microglia activation in a range of activation conditions.

## Discussion

In this work, we describe an automated image analysis method capable of accurately segmenting individual fluorescently labeled microglia from *in situ* image data and calculating morphological parameters that describe their shape, enabling the rapid analysis of large populations. Segmentation is achieved through a novel local iterative threshold method, which relies on a single variable, target cell size. Furthermore, we provide evidence that microglia activation can be quantified *in situ* based on morphometric analysis of microglia; specifically, one parameter, soma size, exhibits an LPS dose-dependent increase concurrent with increasing IBA-1 expression. We also provide proof-of-concept data demonstrating that longitudinal assessment of soma size provides a quantitative means to capture the dynamics of microglia insult response *in vivo* under LPS and thermal-injury activating conditions.

One of the greatest challenges in automatically detecting cell morphology from fluorescence microscopy data is compensating for uneven fluorescence detection across a field of view. This is of particular importance when relying on subtle changes in cell morphology as readout of cell physiology. If background levels vary widely between image regions, applying a global threshold will result in non-uniform identification of cell shape dependent upon where the cell is located in the field of view. In the context of microglia, highly ramified cells, resting cells may appear more rounded, and therefore activated, if a slightly higher intensity threshold is used to segment the image. We overcame this problem by first detecting the positions of the cells within a field of view by utilizing regional maxima finding algorithm. Accurate cell segmentation could then be achieved by applying a local iterative threshold operation within the immediate area surrounding each detected soma. In this manner we could achieve 70–100% accuracy in cell detection and segmentation at ∼1/100^th^ the time required for manual intervention.

The morphometric analysis described above relies on segmentation of two-dimensional projection representing a three dimensional volume. As the distribution of branches from a microglia appears to be random at rest with respect to X, Y and Z dimensions [12], analysis of branching in a three dimensional volume projection should provide a representative estimate of total cell shape and branching pattern. A projection through 10 µm of Z depth, we show, is sufficient to quantitatively assess changes in microglia shape. The Z depth of 10 µm is also of importance as it enables consistent analysis of cell shape and immunostaining within tissue sections where antibody penetration is limited.

The cell segmentation method outlined in the manuscript is directly applicable to defining cell-based ROIs to quantify fluorescent intensities of various different markers. The ability to measure cell shape change is dependent on contrast across the labeled cell, and therefore, will only be compatible with labeling methods that provide staining throughout the cell. Care should be taken to analyze markers that vary in staining intensity with microglia activation, such as IBA-1. This is because activated cells will be easier to detect than resting cells, potentially introducing bias.

Several imaging modalities enable the quantitative measure of microglia activation *in vivo*. Positron emission tomographic imaging of benzodiazepine receptor radioligands, for example, quantitatively report the upregulation of PBR expression induced upon microglia activation [15]. Preclinical magnetic resonance and bioluminescence imaging methods are also emerging for assessing neuroinflammation *in vivo* [16], [17], [18]. While powerful for assessing gliosis on a whole organ basis, these techniques are limited in both spatial resolution and temporal flexibility.

Conversely, the image analysis routines and microscopy techniques utilized in this study enable the quantitative assessment of microglia activation within large regions of the cortex longitudinally *in vivo* with cellular resolution and on timescales of seconds to months. Automated image segmentation provides a rapid unbiased method, enabling analysis of large datasets consisting of numerous imaging volumes and/or multiple time points. Furthermore, our morphometric analysis extends previous microscopy-based studies [8], [19], [20], and supports its use as a quantitative metric of microglia activation.

The methods outlined above should also be directly applicable to the study of microglia biology in other nervous system tissue, or insult conditions; however, the morphological metrics should be recalibrated to match the experiment. For example, the morphology of resting microglia can vary with the nervous system region [12]. Therefore, the morphometric parameters used for assessing activation should be confirmed per region and microglia morphologies compared only within the same region.

Finally, the morphology analysis method here is not limited to the study of microglia. There are other cell types, such as dendritic cells that undergo considerable morphology change with activation [21]. These methods could be used to rationally construct shape-based activation indicators for cell activation time-course *in vivo*.

## Supporting Information

Figure S1
**Convergence of cell size by iterative threshold segmentation.** (**A**) A MIP image of a typical microglia. (**B**) Example of segmented CCM when an estimated intensity threshold is set too high (exaggerated for illustrative purposes). This gives a CCM with a very small area and does not capture the features of the cell. The iterative method ensures that the threshold is modified at each iteration, so that the detected cell size eventually converges* near the TCS** (**C**). (**D**) Similarly, if a threshold is too low initially, the iterative method ensures that the CCM area converges to a similar (in this case identical) value as in (**C**). (**E**). Note that both iterative methods produce segmented images of very similar appearance (compare the last image in **B** to that in **D**). All images are of the same scale. Scale bar equals 50 µm. * Typically, the starting image threshold estimate by the Otsu method using the MATLAB graythresh function generates a CCM closer to the final CM than the thresholds used in this illustration, which are examples of “worst case” scenarios. In order to investigate the robustness of the convergence algorithm under these conditions, we substituted the typical Otsu estimated starting image threshold with either Otsu threshold divided by 2 or multiplied by 2. This is a wide range that should safely encompass any errors that would normally arise from the initial threshold estimation. The mean differences between the cell sizes obtained from these different starting thresholds were computed as:
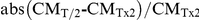
Where CM_T/2_ = cell mask size obtained from using half the Otsu value for a starting threshold; CM_Tx2_ = cell size obtained from using double the Otsu value for a starting threshold. Computed for 10,000 cells, the average value was 8.10%, showing that even in the worst case scenario, the mean error in convergence is relatively small: less than 10%. **For the method used to determine the optimal TCS, see Figure S2.(TIF)

Figure S2 **Dependency of morphometric parameter threshold values.** (**A**) The influence of TCS on morphometric parameters and detected IBA-1 and CD68 expression, for control, 2 mg/kg, and 4 mg/kg doses of LPS in fixed (IHC) samples. TCS value of 500 µm^2^ provided maximal cell detection and the ability to distinguish between LPS conditions based on measured parameters. (**B**) Effect of threshold level on soma size detection in both live and fixed samples. The soma mask is identified utilizing a threshold value x above the last iterative threshold used to segment the entire cell. When x is increased from 0.1 to 0.9, there is a decrease in recorded soma size. However, for whatever value of x, the results delineated between microglia from LPS stimulated, and control samples. This implies that the exact value of x will not influence the ability to differentiate between activated and control microglia.
(TIF)

Figure S3 **Comparisons of manual and automated methods to detect cells.** (**A**) Examples of manual and automatic cell detection of microglia under different LPS conditions 24 h prior to tissue collection. The primary processes were manually scored (red spots). Only cells that are fully in focus (the soma is visible), and not touching the border of the frame or other cells, are counted. The right shows automatically segmented cells in various colors, and white circles that indicate the detected cell bodies. Scale bar equals 50 µm. (**B**) Summary of the time taken, total number of detected cells, the total number of processes detected, and the average number of processes per cell detected by the manual or automatic method. Note that counting processes manually is very difficult, because of the ‘fractal-like’ shape of microglia, where small processes extend from larger processes. Therefore, the manual method must arbitrarily determine the size of a process that is large enough to be counted.
(TIF)

Figure S4 **Distribution of Morphometric Parameters, IBA-1 and CD68 expression per cell in control samples.** Histogram plots of the distribution of morphometric parameters, and IBA-1 and CD68 expression assessed from n = 5 animals under control conditions.
(TIF)

Figure S5 **Per cell quantification of IBA-1 and CD68 expression.** Cell-specific regions of interest defined through segmentation of individual microglia based on EGFP fluorescence (white outline). Protein expression within each ROI is quantified based on fluorescence intensity of the corresponding secondary antibody label before any contrast adjustment. For illustration, the images are contrast adjusted to aide in visualizing the IHC stain. Scale bar equals 50 µm.
(TIF)

Figures S6 **Correlation plots between IBA-1 expression and CD68 expression or morphometric descriptors.** (**A**) Correlation plots between the intensity of IBA-1 and CD68 expression for control, 1, 2, and 4 mg/kg doses. (**B**) Correlation plots for all morphological parameters vs. IBA-1 expression for control, 1 mg/kg, 2 mg/kg, and 4 mg/kg doses. Value indicates the mean linear correlation coefficient (R^2^) for dataset with n = 7, 5, 5 and 6 animals, respectively.
(TIF)

Figure S7 **The influence of resolution in X, Y, and Z axes on segmentation and soma size estimates.** The role of resolution on microglia soma size detection was explored. When the Z step size was changed from 1 µm to 2 µm, or when pixel size was changed from 0.76 µm/pixel to 1.33 µm/pixel, a negligible change in mean soma size was detected. However, individual cells may or may not be segmented depending on conditions as the exact sections used may reveal cell-cell contacts. Scale bar equals 50 µm.
(TIF)

Figure S8 **Soma size tracks microglia activation under laser ablation conditions.** A mouse had cranial window surgery as described in methods, 2 weeks before the experiment, and was injected with AngioSense 680 prior to imaging. Three neighboring 245 µm×245 µm×40 µm (XYZ) volumes (zoom = 1.5, 512×512 pixels spaced 2 µm in Z) were selected, with a single site of focal damage per volume. Pixel size was 0.47 µm/pixel. The zoom was used to limit the analysis area to the region immediately next to the site of ablation. A focused laser beam of approximately 200 mW with a dwell time of 10 µs/pixel was used to irradiate a microglial cell in the center of the imaging area. Successful laser ablation was confirmed by observing microglia processes extending to the damaged area, as in Davalos et al. [22]. (**A**) Fold change in soma size over days after laser ablation on day 0 (immediately after ablation) then at 24 h, 48 h, and 72 h, averaged over the 3 volumes representing 36 individual microglia. (**B**) Blood vessels are filled with red (AngioSense 680) and microglia are in green (EGFP). The border colors on the microglia indicate soma size, red corresponds to microglia with soma size >65 µm^2^, thus activated. Scale bar equals 50 µm.
(TIF)
