# Supplementary figures and images for: An Automated Method to Quantify Microglia Morphology and Application to Monitor Activation State Longitudinally In Vivo
Source: PLoS One. 2012 Feb 28;7(2):e31814. doi: 10.1371/journal.pone.0031814 (PMC3294422; doi:10.1371/journal.pone.0031814)

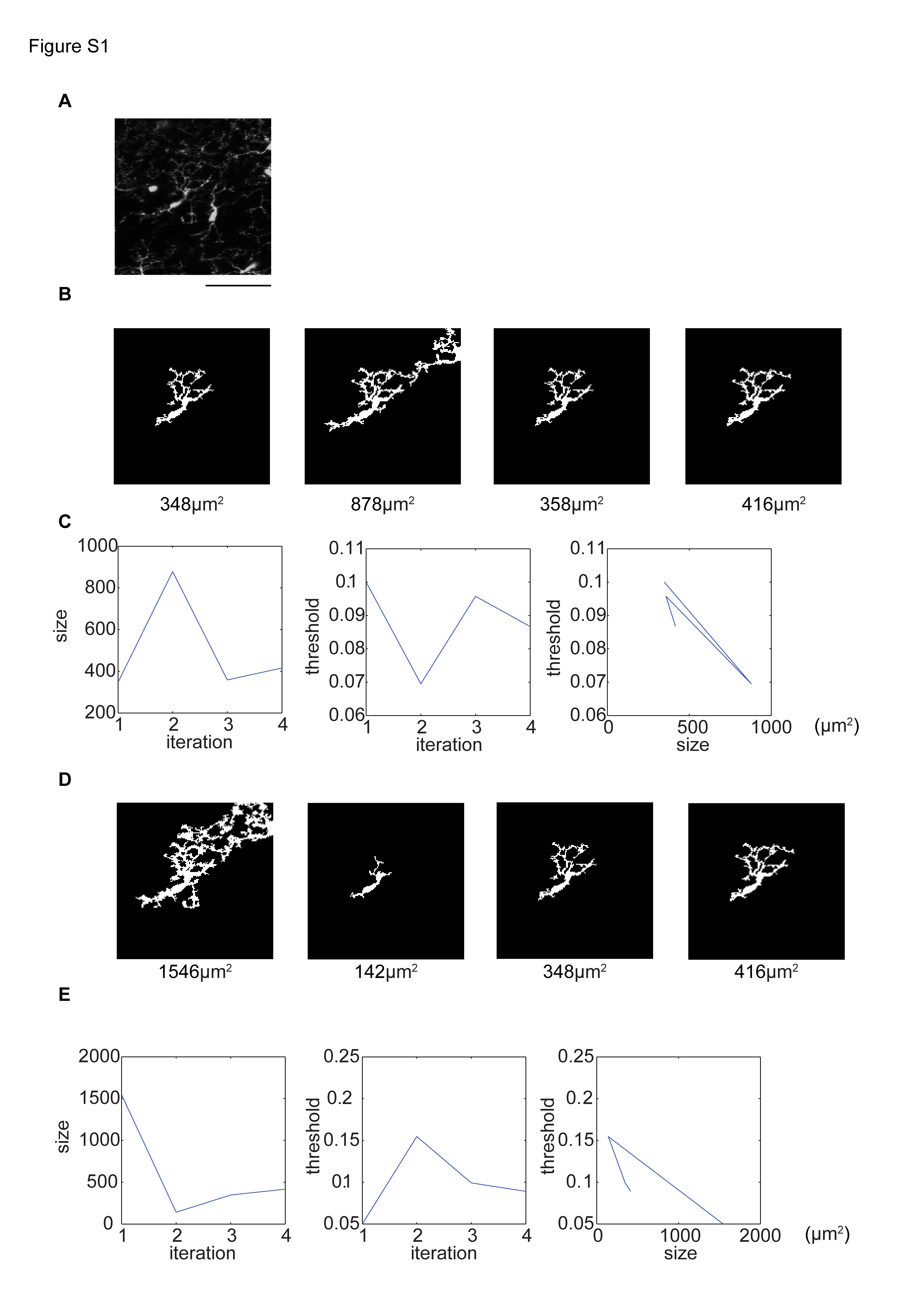

Supplement: Figure S1 — Convergence of cell size by iterative threshold segmentation. (A) A MIP image of a typical microglia. (B) Example of segmented CCM when an estimated intensity threshold is set too high (exaggerated for illustrative purposes). This gives a CCM with a very small area and does not capture the features of the cell. The iterative method ensures that the threshold is modified at each iteration, so that the detected cell size eventually converges* near the TCS** (C). (D) Similarly, if a threshold is too low initially, the iterative method ensures that the CCM area converges to a similar (in this case identical) value as in (C). (E). Note that both iterative methods produce segmented images of very similar appearance (compare the last image in B to that in D). All images are of the same scale. Scale bar equals 50 µm. * Typically, the starting image threshold estimate by the Otsu method using the MATLAB graythresh function generates a CCM closer to the final CM than the thresholds used in this illustration, which are examples of “worst case” scenarios. In order to investigate the robustness of the convergence algorithm under these conditions, we substituted the typical Otsu estimated starting image threshold with either Otsu threshold divided by 2 or multiplied by 2. This is a wide range that should safely encompass any errors that would normally arise from the initial threshold estimation. The mean differences between the cell sizes obtained from these different starting thresholds were computed as:Where CMT/2 = cell mask size obtained from using half the Otsu value for a starting threshold; CMTx2 = cell size obtained from using double the Otsu value for a starting threshold. Computed for 10,000 cells, the average value was 8.10%, showing that even in the worst case scenario, the mean error in convergence is relatively small: less than 10%. **For the method used to determine the optimal TCS, see Figure S2. (TIF) [file pone.0031814.s001.tif]

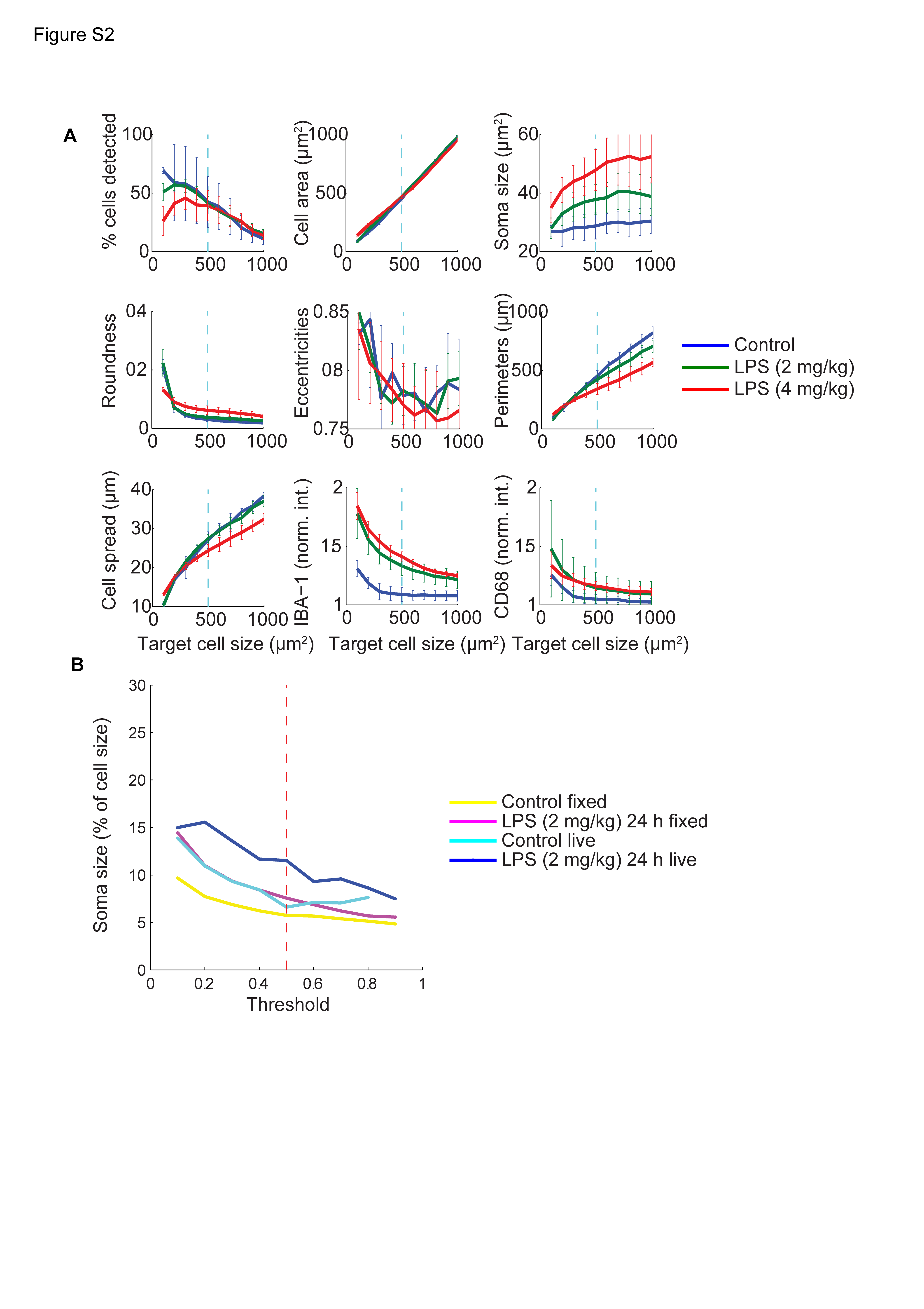

Supplement: Figure S2 — Dependency of morphometric parameter threshold values. (A) The influence of TCS on morphometric parameters and detected IBA-1 and CD68 expression, for control, 2 mg/kg, and 4 mg/kg doses of LPS in fixed (IHC) samples. TCS value of 500 µm2 provided maximal cell detection and the ability to distinguish between LPS conditions based on measured parameters. (B) Effect of threshold level on soma size detection in both live and fixed samples. The soma mask is identified utilizing a threshold value x above the last iterative threshold used to segment the entire cell. When x is increased from 0.1 to 0.9, there is a decrease in recorded soma size. However, for whatever value of x, the results delineated between microglia from LPS stimulated, and control samples. This implies that the exact value of x will not influence the ability to differentiate between activated and control microglia. (TIF) [file pone.0031814.s002.tif]

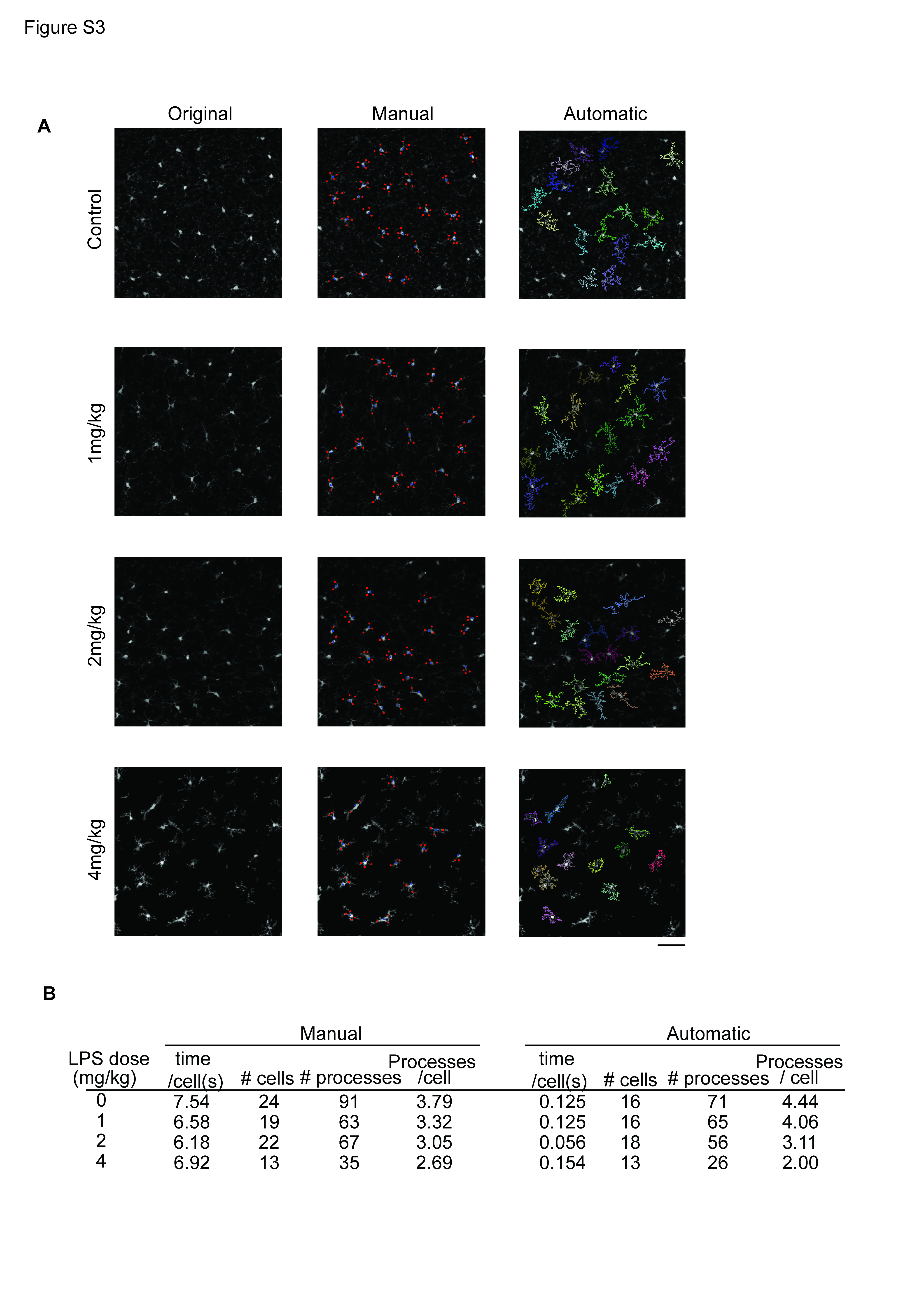

Supplement: Figure S3 — Comparisons of manual and automated methods to detect cells. (A) Examples of manual and automatic cell detection of microglia under different LPS conditions 24 h prior to tissue collection. The primary processes were manually scored (red spots). Only cells that are fully in focus (the soma is visible), and not touching the border of the frame or other cells, are counted. The right shows automatically segmented cells in various colors, and white circles that indicate the detected cell bodies. Scale bar equals 50 µm. (B) Summary of the time taken, total number of detected cells, the total number of processes detected, and the average number of processes per cell detected by the manual or automatic method. Note that counting processes manually is very difficult, because of the ‘fractal-like’ shape of microglia, where small processes extend from larger processes. Therefore, the manual method must arbitrarily determine the size of a process that is large enough to be counted. (TIF) [file pone.0031814.s003.tif]

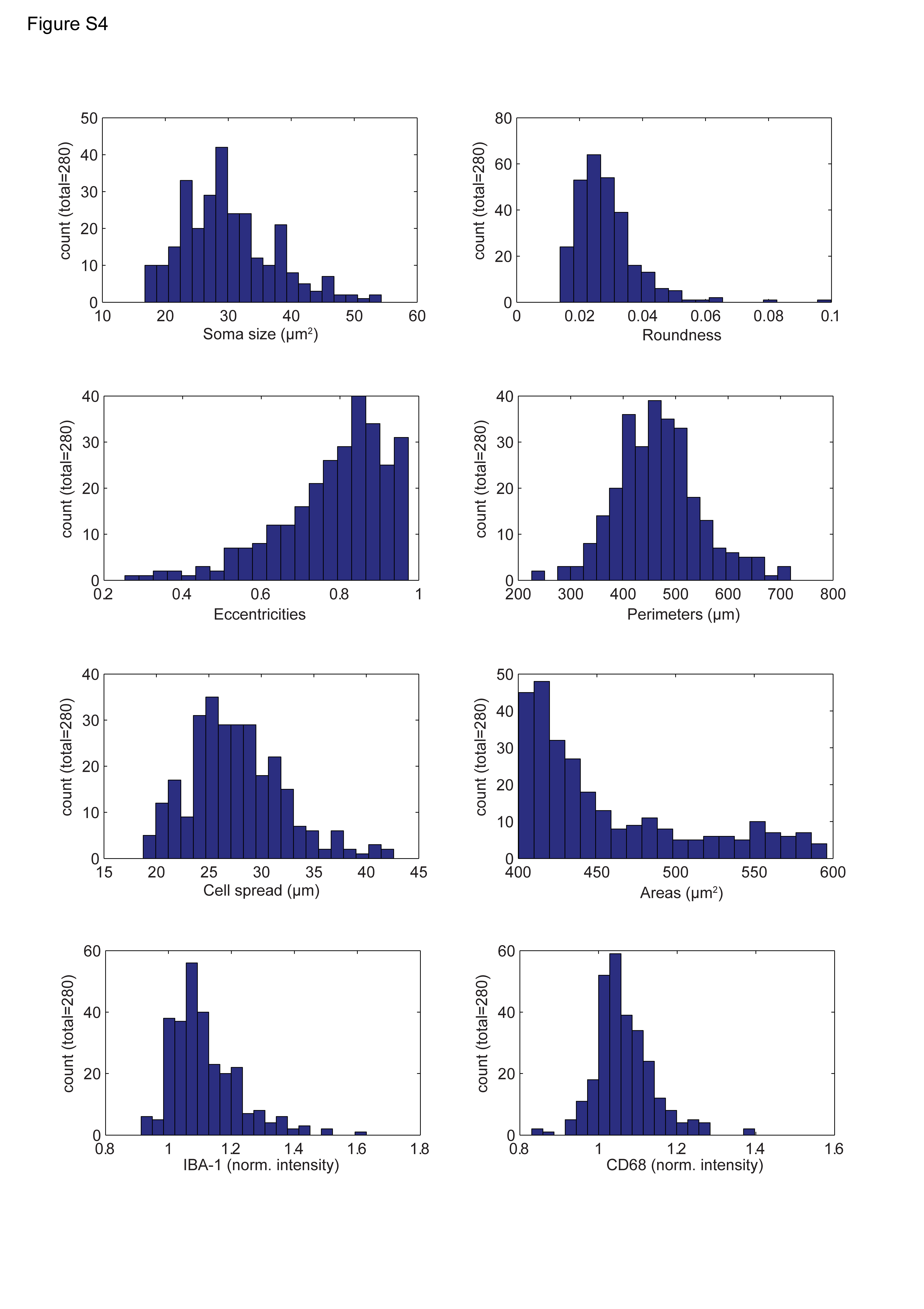

Supplement: Figure S4 — Distribution of Morphometric Parameters, IBA-1 and CD68 expression per cell in control samples. Histogram plots of the distribution of morphometric parameters, and IBA-1 and CD68 expression assessed from n = 5 animals under control conditions. (TIF) [file pone.0031814.s004.tif]

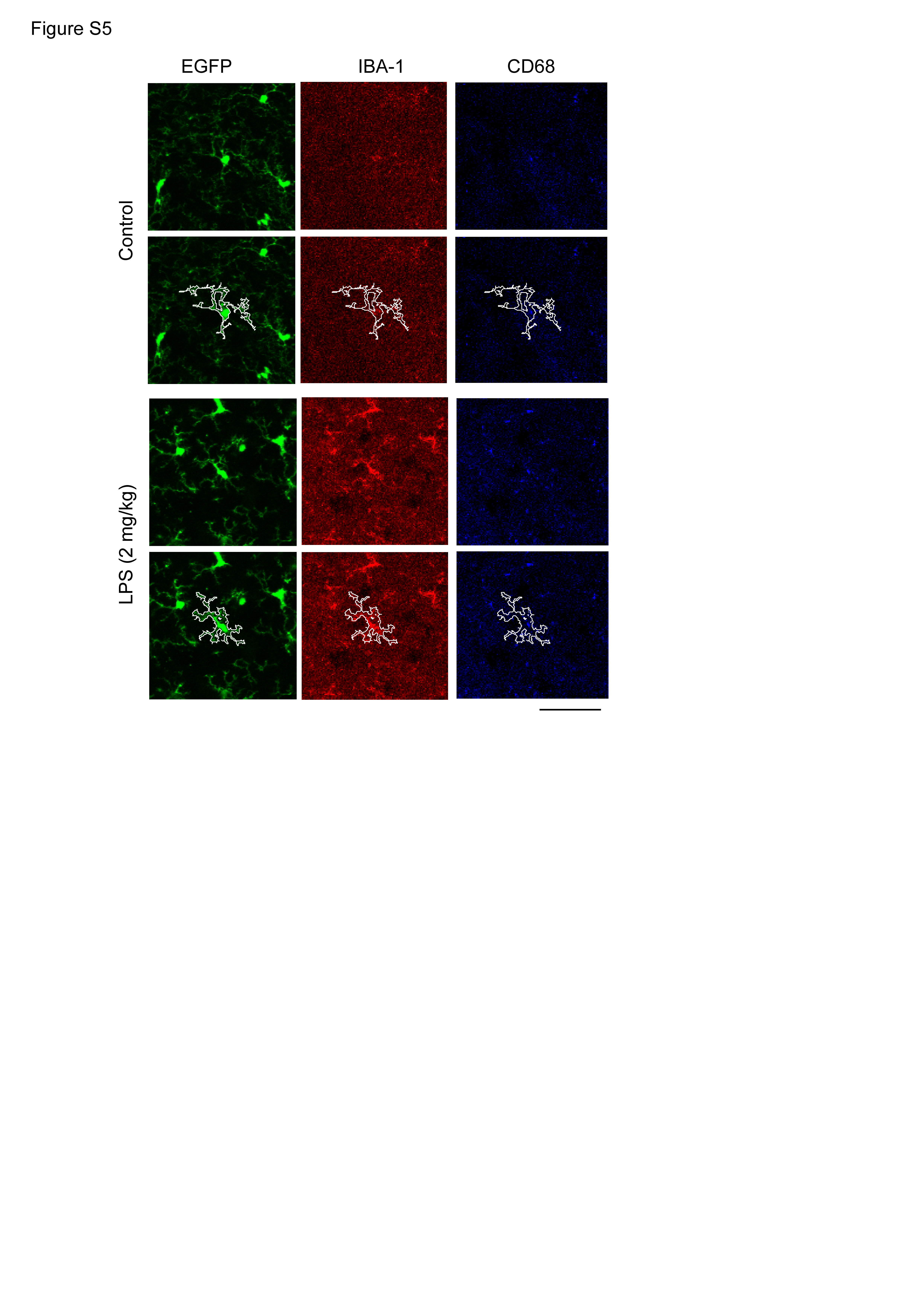

Supplement: Figure S5 — Per cell quantification of IBA-1 and CD68 expression. Cell-specific regions of interest defined through segmentation of individual microglia based on EGFP fluorescence (white outline). Protein expression within each ROI is quantified based on fluorescence intensity of the corresponding secondary antibody label before any contrast adjustment. For illustration, the images are contrast adjusted to aide in visualizing the IHC stain. Scale bar equals 50 µm. (TIF) [file pone.0031814.s005.tif]

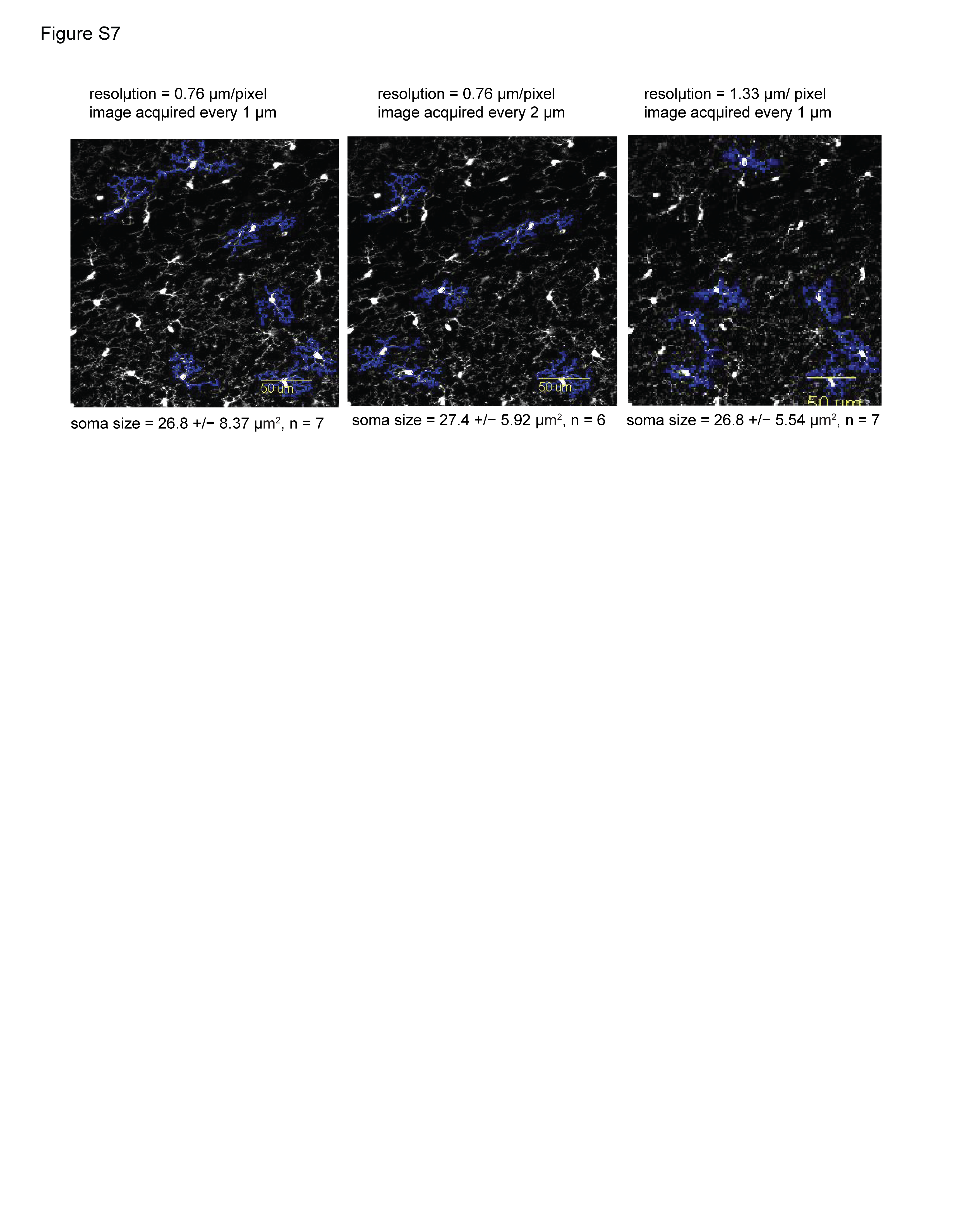

Supplement: Figure S7 — The influence of resolution in X, Y, and Z axes on segmentation and soma size estimates. The role of resolution on microglia soma size detection was explored. When the Z step size was changed from 1 µm to 2 µm, or when pixel size was changed from 0.76 µm/pixel to 1.33 µm/pixel, a negligible change in mean soma size was detected. However, individual cells may or may not be segmented depending on conditions as the exact sections used may reveal cell-cell contacts. Scale bar equals 50 µm. (TIF) [file pone.0031814.s007.tif]

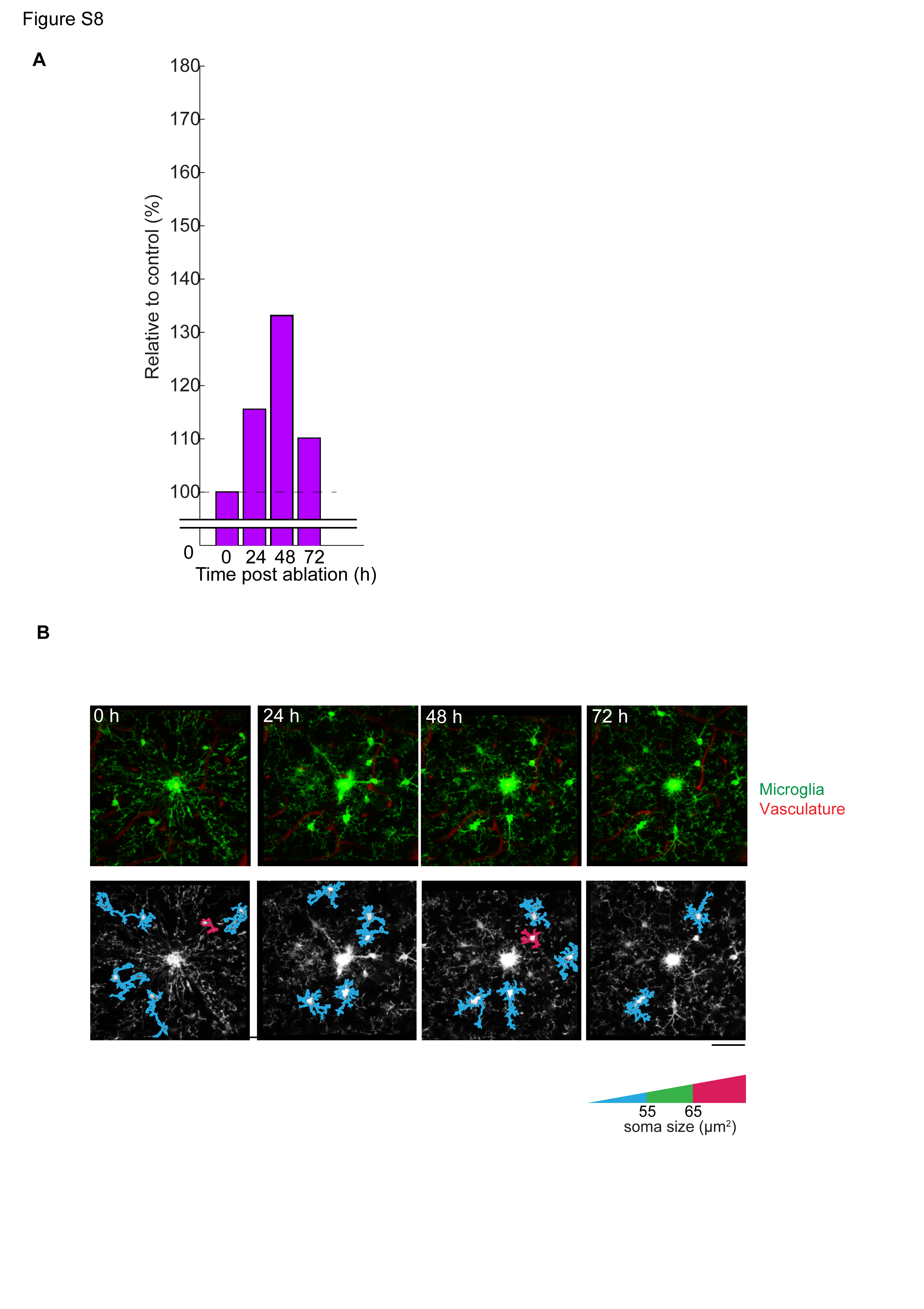

Supplement: Figure S8 — Soma size tracks microglia activation under laser ablation conditions. A mouse had cranial window surgery as described in methods, 2 weeks before the experiment, and was injected with AngioSense 680 prior to imaging. Three neighboring 245 µm×245 µm×40 µm (XYZ) volumes (zoom = 1.5, 512×512 pixels spaced 2 µm in Z) were selected, with a single site of focal damage per volume. Pixel size was 0.47 µm/pixel. The zoom was used to limit the analysis area to the region immediately next to the site of ablation. A focused laser beam of approximately 200 mW with a dwell time of 10 µs/pixel was used to irradiate a microglial cell in the center of the imaging area. Successful laser ablation was confirmed by observing microglia processes extending to the damaged area, as in Davalos et al. [22]. (A) Fold change in soma size over days after laser ablation on day 0 (immediately after ablation) then at 24 h, 48 h, and 72 h, averaged over the 3 volumes representing 36 individual microglia. (B) Blood vessels are filled with red (AngioSense 680) and microglia are in green (EGFP). The border colors on the microglia indicate soma size, red corresponds to microglia with soma size >65 µm2, thus activated. Scale bar equals 50 µm. (TIF) [file pone.0031814.s008.tif]
